# Ginseng Biomacromolecules: Integrating Nutrition and Health, a New Direction in Phytomedicine

**DOI:** 10.3390/ijms27052151

**Published:** 2026-02-25

**Authors:** Ying Liu, Jiawei Li, Chen Chen, Mengyang Wang, Min Zhang, Wei Liu

**Affiliations:** 1 College of Pharmacy, Beihua University, Jilin 132013, China; 13514853284@163.com (Y.L.); 15243205528@139.com (J.L.); chen3535668@outlook.com (C.C.); 15643584357@163.com (M.W.); 2 Jilin Province Ginseng Resources Development and Transformation Key Technology Engineering Laboratory, Beihua University, Jilin 132013, China; 3 College of Basic Medicine, Beihua University, Jilin 132013, China

**Keywords:** ginseng biomacromolecules, structure analysis, biological function, functional food, application prospect

## Abstract

As a traditional dual-purpose ingredient for both medicine and food, the biomacromolecules in *Panax ginseng* include polysaccharides, pectin, exosomes, proteins and dietary fiber. Due to their unique chemical structures, physiological activities, and processing adaptability, these components have achieved diversified applications in the medical field, becoming one of the core raw materials for functional food development. Modern research shows that the biomacromolecules found in ginseng can regulate the body’s immunity, antioxidant and anti-tumor properties, as well as antibacterial properties and the ability to enhance the body’s metabolic capacity, demonstrating significant application potential in healthcare-related fields. Recent studies have found that in addition to the root, the stems, leaves, fruits and flowers of *P. ginseng* also contain various effective components such as ginseng polysaccharides and pectin, which have enhanced the utilization value of ginseng plant resources. Ginseng biomacromolecules can not only replace antibiotics but also improve the production performance of animals by influencing the structure of intestinal flora, providing raw materials for the selection and application of natural feed additives for animals. This review summarizes the latest research findings on the pharmacological properties and practical applications of ginseng-derived biomolecules. It primarily addresses the structural characteristics, pharmacological activities, and current applications in health and medicine of biomolecules such as ginseng polysaccharides, ginseng exosomes, ginseng proteins, and ginseng dietary fiber. It aims to provide a fresh perspective and a solid theoretical foundation for the in-depth development of ginseng in the fields of medicine and molecular biology.

## 1. Introduction

*Panax ginseng* C. A. Meyer is an important medicinal plant resource in Asia and even in the world. It occupies an important position in traditional Chinese medicine (TCM), including a series of bioactive chemicals that play a key role in promoting health and treating diseases [1]. Studies have shown that *P. ginseng* has a wide range of pharmacological effects. These include enhancing immune function, antioxidant activity, and tumor growth inhibition, which are attributed to its diverse bioactive components. These effects are partly attributed to various biological substances found in ginseng, including acidic polysaccharides [2], exosomes [3], and others. These substances play a crucial role in regulating the body’s immunity, promoting blood circulation, and enhancing health. They also serve as functional factors enabling ginseng’s biological activity, clinical drug development, and functional food innovation, forming the cornerstone for advancing ginseng’s modern medical and health applications.

Ginseng polysaccharides are the second most important active compounds in *P. ginseng* after ginsenosides, and they also serve as the core components of ginseng macromolecules [4]. With the development of extraction and separation technologies, the application of biomacromolecules such as ginseng polysaccharides has become increasingly widespread. Ginseng biomacromolecules constitute a crucial class of bioactive components within ginseng, serving as the fundamental material basis for its medicinal and nutritional value [5]. Modern research has revealed that ginseng polysaccharides, pectins, proteins, and exosomes exhibit significant anti-cancer properties, immune-boosting effects, anti-aging benefits, and lipid metabolism regulation capabilities [5,6]. As studies deepen, it has become clear that the biological activity of ginseng macromolecules is closely linked to the composition of polysaccharides, the spatial structure of proteins, and the binding sites of glycosidic bonds. How to leverage the potential value of these ginseng macromolecules represents a major focus in current ginseng research.

Previous studies have conducted comprehensive analyses of active components, such as ginsenosides and polypeptides in ginseng, clarifying their potential value in promoting health and combating diseases [7,8]. In recent years, research on the biological substances in ginseng, such as polysaccharides, pectin, exosomes, proteins, and dietary fibers, has been gradually increasing. These studies have underscored their pharmacological significance [9]. The pharmacological activities of these biological substances highlight their importance in the functional food sector. Additionally, the biomolecules in ginseng have been incorporated into functional candies, baked goods, health foods, and other products. These biomolecules not only enrich the nutritional profile of foods but also enhance product value by leveraging ginseng’s traditional efficacy and nutritional benefits [10]. Further exploration of their properties and activities will advance the modernization of TCM and benefit global health.

**Figure 1:**
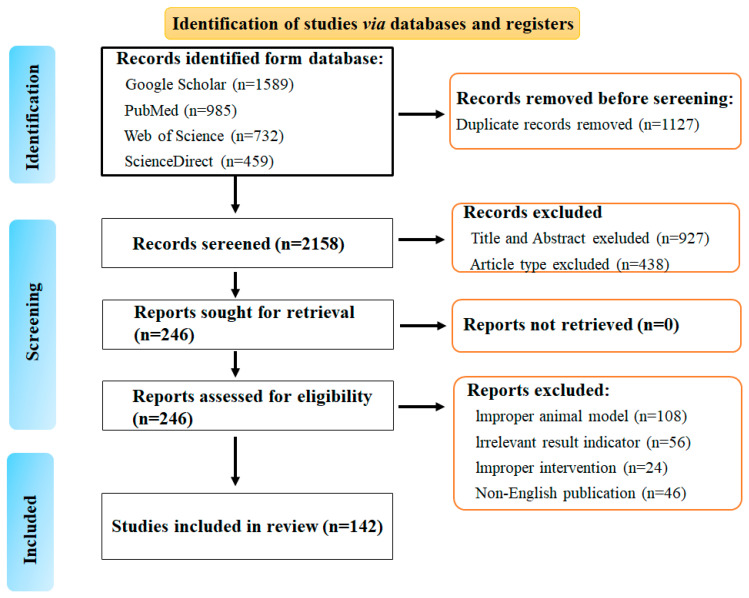
PRISMA flowchart for literature screening.

Relevant literature was systematically searched in PubMed, Web of Science, Google scholar and ScienceDirect databases. Search terms included antitumor, anti-cancer, pharmacological effects, antidiabetic, anti-aging, ginseng polysaccharides, ginseng pectin, ginseng proteins, ginseng exosomes, ginseng dietary fiber, and ginseng biomacromolecules. Eligible studies encompassed research on the biological activity, pharmacological effects, and applications of ginseng polysaccharides, ginseng pectin, ginseng proteins, ginseng exosomes, ginseng dietary fiber, or ginseng biomacromolecules in vitro and in vivo experimental models. Results addressed the structural characteristics, research progress, and potential molecular mechanisms of ginseng macromolecules, such as antioxidant effects, apoptosis, lipid metabolism, inflammation, anti-aging, angiogenesis, metastasis, and immunomodulation. Studies involving ginsenosides, amino acids, compound formulations, non-English publications, and duplicate records were excluded. This review adheres to the PRISMA guidelines, with the literature screening process illustrated in Figure 1.

This review aims to summarize research progress on ginseng biomacromolecules in the food sector, focusing on their structural characteristics, biological activities, and application strategies in food. It also deepens our understanding of ginseng’s mechanisms of action across various health conditions while advancing its research and application expansion, providing crucial references for promoting ginseng’s widespread use in modern medicine and health fields.

## 2. The Current Application Status of Ginseng Polysaccharides and Pectin

Ginseng polysaccharides are important active components in *P. ginseng*. They are complex biomacromolecules formed by the connection of arabinose (Ara), galactose (Gal), rhamnose (Rha), galacturonic acid (Gal A) and glucuronic acid (Glu A) through glycosidic bonds, with a molecular weight range of 3.5 × 10^3^~2.0 × 10^6^ Da [10,11]. They mainly contain neutral sugars and acidic sugars, and the active component is mainly acidic pectin. Studies have confirmed that the roots, stems, leaves, flowers and fruits of ginseng all contain polysaccharides, which possess biological activities such as anti-cancer, anti-inflammatory, anti-aging, immune regulation and enhancing the body’s metabolism. Even some of their pharmacological effects are superior to those of ginsenosides, and they have broad application potential [4].

### 2.1. The Physicochemical Properties of Ginseng Polysaccharides

**Figure 2:**
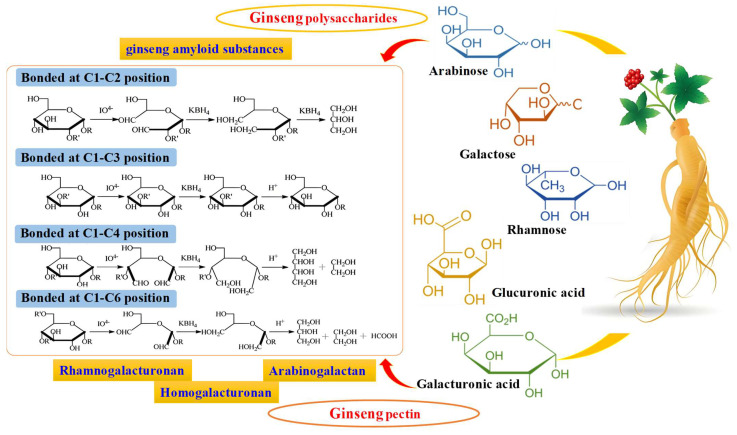
Sugar chain arrangement and monosaccharide composition of polysaccharides and pectins in ginseng biomacromolecules.

The polysaccharide content of ginseng is approximately 5%, mainly composed of ginseng amyloid substances and ginseng pectin. Among them, ginseng amyloid substances account for approximately 80%, with the main component being amyloid glucan, which is composed of α -*D*–(1,4) -glucan, 6-branched α -*D*–(1,4) -glucan, 3-branched α -*D*–(1,6) -glucan, and α -*D*–(1,6) -glucan without side chains [11] (Figure 2). Ginseng pectin accounts for approximately 20% and is the main active substance of ginseng polysaccharides. It is an acidic heteropolysaccharide mixture mainly composed of Gal, Gal A, Rha, arabinoxylan (AX), etc., forming three structural domains: rhamnogalacturonans (RG), homogalacturonan (HG), and arabinogalactan (AG) [12].

The RG components in ginseng pectin primarily consist of two types: RG-I and RG-II. Among these, RG-I is the most extensively studied domain and is most closely associated with biological activity. Its backbone primarily consists of alternating α-1,2-linked Rha and α-1,4-linked Gal A units. Side chains of galactan, arabinan, and AG are attached to the Rha residues [5]. Modern research has found that the composition and length of the RG side chain have a significant impact on the biological activity of RG-I [11]. The AG side chain in RG-I is a necessary structure for stimulating macrophage secretion of nitric oxide and promoting lymphocyte proliferation [9]. Moreover, the ginseng pectin fragment rich in RG-I (such as RG-I-4) can effectively inhibit the adhesion of cancer cells and the binding of the immune checkpoint protein galectin-3 (Gal-3) to T cells, exerting anti-tumor effects [10,12]. HG is formed by the linear connection of D-Gal A through α-1,4-glycosidic bonds. It is one of the main chains of ginseng pectin and accounts for approximately 10% of ginseng polysaccharides. It is a crucial component for maintaining the molecular framework and physical properties. Furthermore, the biological activity of the HG domain is closely related to the degree of esterification (DE). The carboxyl group at the C-6 position of the Gal A residue can be esterified, which affects the solubility, gelation ability and interaction with immune cells of pectin.

RG-I-4 is an RG-I domain purified from ginseng pectin using DEAE-cellulose ion exchange chromatography and enzymatic hydrolysis. It primarily consists of GalA, Glc, Ara, Gal, and Rha [13]. Research has found that the structure of water-soluble ginseng pectin (WGPA-2-RG) isolated from ginseng polysaccharides, with a molecular weight of approximately 1 × 10^3^ Da, mainly consists of the pectin fraction with the AG domain structure. The AG side chains in its structure are essential for stimulating the secretion of nitric oxide and the proliferation of lymphocytes [14]. These structural characteristics can finely regulate the polarity and charge distribution of molecules and the interactions between biomolecules, activating specific signaling systems. Meantime, the spatial conformation and chemical modification of WGPA-2-RG enhance its anti-tumor efficacy through precise molecular engineering in synergy. The study found that after removal of Ara and Gal residues from in the polysaccharide domains (RG-I-4 and WGPA-2-RG) of ginseng pectin through hydrolysis, the phagocytic promoting effect on macrophages did not change [15]. This suggests that Ara and Gal may not be the key structural units responsible for the polysaccharide’s immune-activating function. Instead, its phagocytosis-promoting effect likely depends on the polysaccharide’s backbone configuration, acetyl groups, RG-regions, or overall spatial conformation. This finding provides important clues for further elucidating the structural basis of ginseng pectin’s immune activity, suggesting that subsequent studies may focus on the mechanisms of action of core functional domains or modified groups that remain intact after hydrolysis [16].

Additionally, ginseng polysaccharide exhibits various biological activities, including immune system regulation [17,18,19], inhibition of tumor cell migration, antioxidant effects, and others. These activities highlight the potential of ginseng polysaccharide in promoting health and provide a scientific basis for understanding the overall medicinal value of P. ginseng. The main functions related to ginseng polysaccharide are summarized in (Table 1). However, due to the high molecular weight of ginseng polysaccharides, it is very difficult to clarify the entire structure using chemical and spectral techniques. Therefore, the structure-activity relationship is not yet fully clear, and a large amount of research is still needed in the future to determine the structure of ginseng polysaccharides [20].

### 2.2. Biological Function of Ginseng Polysaccharides and Pectin

Pharmacological studies have shown that ginseng polysaccharides mainly exert positive and negative regulatory effects on the immune system of the body by regulating immune cells and immune factors, and also possess various activities such as anti-cancer, anti-diabetic and antioxidant properties [27,28]. In addition, ginseng polysaccharides can enhance the body’s metabolism by increasing intestinal absorption and influencing the metabolism of intestinal microorganisms, and have multiple biological effects such as repairing intestinal mucosa, treating ulcerative colitis (UC) [29] and non-alcoholic fatty liver disease (NAFLD) [30,31].

#### 2.2.1. Immunoregulatory Activity

Ginseng pectin, as an important acidic polysaccharide, has shown a multi-dimensional mechanism of action in immune regulation [32,33]. Kim et al. [34] found that ginseng undergoes enhanced immunomodulatory activity following solid-state fermentation with ganoderma lucidum mycelium (GL). The isolated ginseng polysaccharides demonstrated over 70% greater activity than non-fermented ginseng polysaccharides (NG-CP) in promoting macrophage activity, anti-inflammatory effects, and intestinal immune regulation. Gao et al. [35] confirmed that acidic polysaccharides (GL-PI, GL-PII, and GL-PIV) isolated from ginseng stems and leaves exhibit anti-complement activity, which provided a structural basis for its immunomodulatory effects. In addition, red ginseng acidic polysaccharide can activate the TLR2 receptor, trigger the ERK/JNK signaling pathway, and then activate the nuclear transcription factors NF-κB and AP-1, up-regulate the expression of iNOS and the production of nitric oxide and thus enhance the phagocytosis activity of macrophages [36]. Ginseng polysaccharides can also directly activate monocytes and THP-1 cells, promote the production of IL-8, and then recruit neutrophils and T cells to the site of inflammation to enhance anti-infection [37]. In the experimental autoimmune encephalomyelitis (EAE) model, ginseng acidic polysaccharide plays a therapeutic role by inhibiting the proliferation of autoreactive T cells and the production of inflammatory cytokines, and promoting the generation of regulatory T cells, especially by activating the transcription factor Foxp3 [38]. These results indicate that ginseng pectin and its related acidic polysaccharides regulate the immune response through various mechanisms, enhance the immune defense ability of the body, and show potential application value in the treatment of autoimmune diseases.

#### 2.2.2. Anti-Tumor Activity

Research on the anti-tumor effects of ginseng polysaccharides has gradually increased in recent years. Relevant literature indicates that ginseng polysaccharides inhibit various types of cancer cells, including those of lung, liver, and breast cancer [26,39]. A series of studies indicated that ginseng polysaccharides exerts anti-tumor effects through various mechanisms, including the induction of apoptosis [40,41,42], inhibition of cell migration [23], and regulation of tumor necrosis factor (TNF-α) [43]. For instance, Cheng et al. [40] found that ginseng pectin rich in mannose inhibits the proliferation of human colorectal adenocarcinoma cells (HT-29) and induces cell cycle arrest at the G2/M phase. The anti-proliferative effect was significantly enhanced after heat treatment and also promoted caspase-3 activation. Xue et al. [44] confirmed that ginseng pectin specifically inhibits Gal-3-induced T-cell apoptosis, potentially through PKC/ERK and ROS/ERK pathways. In vivo studies also demonstrated ginseng’s efficacy in suppressing Sarcoma-180 tumor growth in mice while promoting T-cell proliferation and IL-2 expression. More importantly, the combination of HG-type ginseng pectin with paclitaxel significantly enhances antitumor efficacy with high safety [24]. The aforementioned studies conclusively demonstrate the synergistic effects of ginseng polysaccharides with chemotherapeutic agents [45,46,47] opening new avenues for tumor prevention and treatment while revealing application strategies for ginseng polysaccharides in antitumor therapy. However, due to the structural complexity of ginseng polysaccharides, future research should focus on elucidating the structure-activity relationships underlying their antitumor effects and clarifying the molecular mechanisms of action to provide practical and effective therapeutic strategies for clinical anti-cancer drugs.

#### 2.2.3. Antioxidant and Anti-Aging Activity

Research indicates that ginseng polysaccharides exert antioxidant effects through mechanisms such as scavenging free radicals, enhancing antioxidant enzyme activity, and inhibiting oxidative stress signaling pathways [21]. Furthermore, antioxidant activity is closely correlated with the molecular weight of the polysaccharides. When molecular weight decreases, the antioxidant action of ginseng polysaccharides primarily occurs via free radical scavenging. Furthermore, the antioxidant activity of ginseng polysaccharides is associated with galactosamine (GalA) residues on their side chains, a characteristic linked to oxidative stress-induced aging. Wang et al. [48] demonstrated that acidic polysaccharides extracted from ginseng exhibit significant anti-aging effects, closely associated with inhibiting ROS production, counteracting oxidative stress damage, and regulating the FOXO/DAF-16 and Nrf2/SKN-1 pathways. These findings confirm the potent antioxidant and anti-aging activity of ginseng polysaccharides, though the precise mechanisms underlying their anti-aging effects warrant further investigation.

#### 2.2.4. Regulation of Glucose and Lipid Metabolism

**Figure 3:**
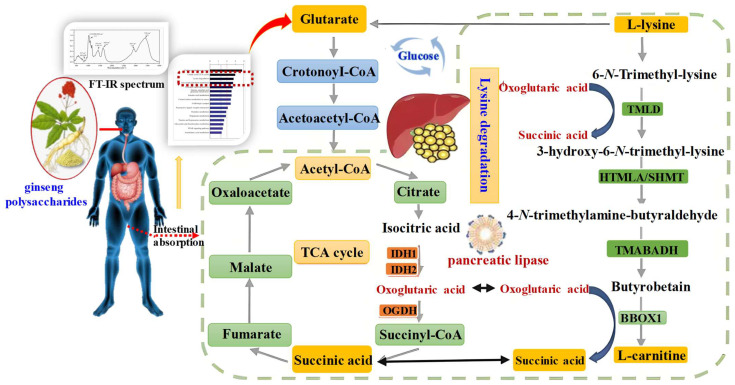
Schematic diagram of the therapeutic potential of ginseng polysaccharides in regulating glucose and lipid metabolism (Lysine degradation and glycolysis).

Previous studies have demonstrated that ginseng polysaccharides regulate glucose and lipid metabolism. Sun et al. [49] established a high-fat diet-induced non-alcoholic fatty liver disease (NAFLD) mouse model, showing that ginseng polysaccharides (WGPA) can modulate carbohydrate digestion and absorption, fatty acid biosynthesis, the pentose phosphate pathway, and fatty acid metabolism. This improves hepatic metabolic dysfunction and prevents NAFLD [50,51]. Furthermore, this process is also associated with gut microbiota dysbiosis [52], alterations in short-chain fatty acids (SCFAs) and insulin resistance [53]. The pathways by which ginseng polysaccharides regulate lipid metabolism are shown in Figure 3. Research has confirmed that ginseng polysaccharides can target and regulate glucose and lipid metabolism, activate the AMPK pathway, increase hepatic glycogen content, and promote lipolysis [54]. This improves lipid metabolism disorders in diabetic and obese rats, laying the foundation for the development of clinically relevant drugs. These studies indicate that ginseng polysaccharides offer novel insights for dietary and therapeutic interventions in diabetes management. However, the glucose and lipid metabolism activity of ginseng polysaccharides varies depending on the methods used for their separation and purification. Therefore, future research should focus on structure-activity relationships, and it is necessary to conduct relevant clinical trials to comprehensively explore its detailed mechanism and therapeutic effect, and propose more effective clinical application strategies.

#### 2.2.5. Other Biological Activities

Ginseng polysaccharides, as key bioactive substances regulating systemic functions, exhibit not only the aforementioned biological activities, but also anti-inflammatory [55], fatigue-relieving [56,57,58], neuroprotective [59], antiviral [60], radiation-protective [61,62], anti-depressant [63], and antibacterial properties [12,64]. Currently, pharmacokinetic studies of ginseng polysaccharides have primarily focused on experimental animal models. Previous researchers employed FITC fluorescent labeling combined with HPLC-MS/MS technology to systematically elucidate the pharmacokinetic characteristics in rats. When administered orally via gavage, ginseng acidic polysaccharides demonstrated significantly superior absorption compared to neutral polysaccharides, whereas no difference was observed with intravenous administration. Tissue distribution exhibited high targeting, with predominant accumulation in the kidneys, liver, and reproductive organs [50,57]. Furthermore, ginseng acidic polysaccharides demonstrate significant anti-diabetic retinopathy and intestinal protective effects in vivo, showing great potential for application in mitochondrial function and intestinal mucosal barrier function [65]. In summary, ginseng polysaccharides possess diverse pharmacological activities with high safety profiles, leading to expanding clinical applications. Their anti-tumor and anti-diabetic effects are particularly prominent, making them a key focus of modern research. However, clinical drug development based on these polysaccharides remains limited, necessitating further studies to elucidate their mechanisms of action.

### 2.3. Application Strategies for Ginseng Polysaccharides and Pectin in Related Fields

Ginseng polysaccharides are natural bioactive compounds, which exhibit significant pharmacological effects and relatively high safety profiles, exhibiting minimal adverse reactions. Consequently, they are highly suitable for extensive application in pharmaceuticals and functional foods. Notably, due to their excellent biocompatibility, ginseng polysaccharides have been demonstrated to serve as effective drug carriers, finding promising applications in the development of food additives and vaccine adjuvants [66]. In addition, ginseng polysaccharides also can be used as natural thickening agents and stabilizers, and have strong application value in food development. However, it is important to note that current research still has certain limitations and remains in the preclinical stage, with insufficient clinical validation. At the same time, the stems, leaves and fruits of ginseng are rich in ginseng pectin, which has development value in animal food additives and poultry production [67]. This sustainable approach embodies environmental protection principles while also opening new avenues for innovation and development in the food industry, contributing to the construction of a green economy [68].

Ginseng polysaccharides possess both water solubility and biocompatibility. They not only serve as natural thickeners and stabilizers to enhance food texture, imparting a smoother mouthfeel to beverages, yogurt, and similar products, but also confer functional properties such as immune modulation and antioxidant effects. This is achieved by regulating gut microbiota balance and boosting immune cell activity. Studies have found that ginseng polysaccharides can also be applied in animal production, not only improve the growth performance of broilers and the production performance of laying hens, but also have the functions of reducing the diarrhea rate and mortality rate of poultry and serving as an adjuvant for vaccines [69]. In addition, adding ginseng polysaccharides to the diet can also be conducive to intensive pig farming, improve the production performance of pigs, and reduce their reliance on antibiotics [70]. Therefore, the comprehensive utilization of ginseng polysaccharides and its by-products is not only economically significant but also contributes to environmental benefits, demonstrating practical value in a win-win situation.

## 3. The Current Application Status of Ginseng Exosomes

Plant exosomes are nanovesicles secreted by plant cells [71], enriched with a diverse array of bioactive molecules that serve as critical mediators of intercellular communication. They encompass DNA, small RNA, proteins, and lipids, all playing essential roles in intercellular communication. Additionally, processing via nanovesicle technology significantly enhances their stability and bioavailability in food processing. Ginseng exosomes share these characteristics, facilitating not only intrinsic signaling but also potentially playing a pivotal role in the interplay between food processing and biological functions. Notably, the biomolecules within ginseng exosomes are closely linked to the plant’s pharmacological activity, opening new avenues for researchers and providing a clear framework for exploring ginseng’s therapeutic potential and underlying mechanisms. Through in-depth investigation of ginseng exosomes and their bioinformation molecules, we may elucidate ginseng’s traditional medicinal value and develop innovative exosome therapies or health foods. This research field holds significant theoretical implications for both plant biology and pharmacology.

### 3.1. Extraction and Characterization Techniques of Ginseng Exosomes

**Figure 4:**
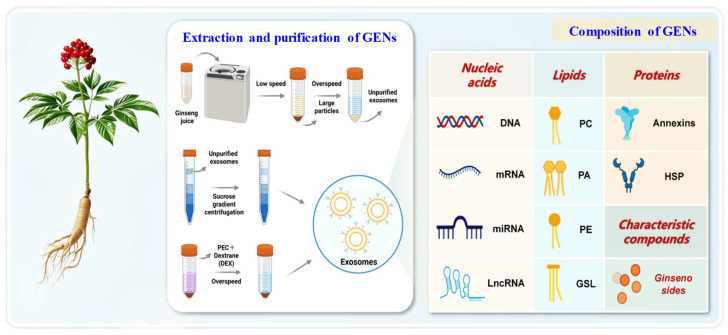
Extraction, Purification, and Molecular Composition of GENs. GEN isolation from fresh ginseng juice. Purified GENs contain diverse bioactive components: nucleic acids (DNA, miRNA, siRNA, mRNA, lncRNA); lipids including phosphatidylcholine (PC), phosphatidic acid (PA), and phosphatidylethanolamine (PE); proteins such as annexins, heat shock proteins (HSPs); and characteristic ginseng metabolites like ginsenosides.

Currently, multiple methodological approaches are employed for the extraction of ginseng exosomes. These include differential centrifugation and ultracentrifugation–ExoQuick combination method, primarily utilizing a series of gradient centrifugation steps [71,72]. Low-speed centrifugation at 2000× *g* removes cellular debris, followed by high-speed centrifugation to isolate exosomes. Additionally, ginseng exosome purification can be enhanced by integrating sucrose density gradient centrifugation. This technique offers relative simplicity and cost-effectiveness; however, it may compromise fragile exosomal membrane proteins during processing, potentially affecting functional integrity in subsequent applications. The functional properties of ginseng exosomes are closely linked to their intricate composition, which includes lipids, proteins, nucleic acids, and other characteristic compounds [72]. The nucleic acids carried by extracellular vesicles can regulate the gene expression of recipient cells (Figure 4). ginseng exosomes mainly contain various types of DNA [73], mRNA, miRNA, etc. [74]. Among lipid constituents, phosphatidylcholine (PC) represents approximately 50% of the exosomal membrane lipids. In conjunction with sphingomyelin, phosphatidylcholine contributes to forming a robust bilayer structure, enhancing resistance to digestive enzyme degradation and ensuring extracellular vesicles stability and activity in complex physiological environments [75,76]. More importantly, ginseng exosomes also contain polysaccharides and small molecule metabolites. The interactions among these components provide a solid biological foundation for the various biological functions of the extracellular vesicles, such as intercellular communication and immune regulation.

### 3.2. Biological Function of Ginseng Exosomes

#### 3.2.1. Anti-Cancer Effect

Modern research has revealed that ginseng exosomes enhance the body’s immune response by activating T cells and macrophages, thereby improving resistance to pathogens. This immunomodulatory effect plays a crucial role in tumor therapy [77]. In oncology, ginseng exosomes have been explored for their anti-tumor mechanisms, primarily through modulation of the tumor microenvironment (TME) [78] One principal mechanism involves activation of the mTOR–T-bet signaling axis via suppression of macrophage-derived ARG1. This modulation reduces T cell exhaustion and enhances the cytotoxic function of CD8+ T cells. In the MC38 colon cancer model, ginseng exosomes treatment has been shown to suppress tumor progression and improve immune surveillance within the TME [79]. At the same time, ginseng exosomes indirectly affect systemic immune homeostasis by regulating gut microbiota composition [80]. This dual regulatory effect—direct immune activation and microenvironment remodeling—offers novel insights for applications in immune-related diseases such as cancer and chronic infections [72]. Moreover, ginseng exosomes can act synergistically with conventional chemotherapeutic agents. Ginseng exosomes–cisplatin co-delivery system has demonstrated enhanced anti-cancer efficacy by targeting tumor cells, inhibiting proliferation and migration, and promoting apoptosis. This combination strategy highlights the potential of ginseng exosomes to improve therapeutic outcomes in chemoresistant tumors [81].

#### 3.2.2. Anti-Inflammatory and Neuroprotective Effects

In immunological contexts, ginseng exosomes exert regulatory effects by modulating immune cell function [82]. One primary mechanism involves regulating macrophage polarization—a critical determinant in the inflammatory response [83]. ginseng exosomes can suppress pro-inflammatory signaling pathways, particularly the TLR4/NF-κB axis. By inhibiting this pathway, ginseng exosomes reduce the production of pro-inflammatory cytokines, such as TNF-α and IL-6, while concurrently enhancing the secretion of the anti-inflammatory cytokine IL-10 [84]. This dual regulatory effect contributes to immune homeostasis and mitigates excessive inflammatory responses.

Furthermore, modern research has revealed that the anti-inflammatory activity of ginseng exosomes is closely associated with neuroprotection. Ginseng exosomes can effectively delay the progression of neurodegenerative diseases such as Alzheimer’s and Parkinson’s [85]. This effect is achieved by suppressing neuroinflammation and oxidative stress while protecting neuronal function. Simultaneously, they further enhance the capacity for neural regeneration, demonstrating their therapeutic potential in neurological disorders. Current research requires further clarification of specific molecular mechanisms, such as the targets of particular miRNAs within exosomes and their role in neural differentiation. However, existing evidence indicates that ginseng exosomes provide a comprehensive therapeutic strategy for multisystem diseases by synergistically exerting anti-inflammatory, repair-promoting, and neuroprotective functions.

#### 3.2.3. Antioxidant Activity

**Figure 5:**
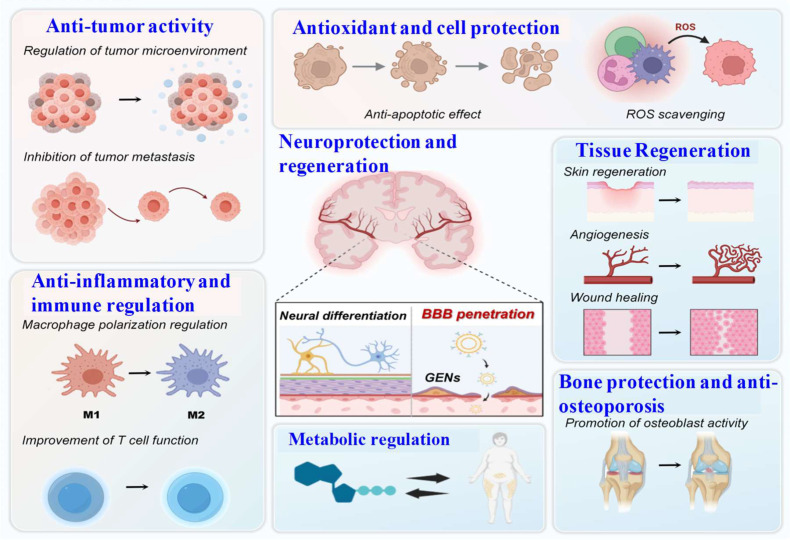
Multifaceted pharmacological activities of GENs. Neuroprotection and regeneration: GENs enhance neural stem cell differentiation and BBB penetration for neurorestorative cargo delivery; Anti-inflammatory and immune modulation: GENs mediate macrophage M1/M2 phenotype transition and improve T-cell responses; Anti-tumor activity: GENs remodel immunosuppressive tumor microenvironments and promote tumor cell apoptosis; Antioxidant activity: Neutralization of reactive oxygen species (ROS); Tissue regeneration: Activation of dermal repair pathways, induction of angiogenesis, and acceleration of epithelialization and tissue remodeling; Bone protection and anti-osteoporosis: Promotion of osteoblast differentiation and mineralization; Metabolic regulation: Modulation of oxidative stress and metabolic balance.

Ginseng exosomes serve as one of the primary carriers of active components, exerting antioxidant effects by scavenging free radicals and mitigating oxidative stress damage [86]. Choi et al. [87] reported that ginseng exosomes can significantly reduce the level of reactive oxygen species (ROS) within cells and effectively alleviate oxidative damage caused by the accumulation of ROS. This property holds considerable promise for applications in anti-aging and the prevention of age-related diseases [88]. Furthermore, studies have confirmed that ginseng exosomes exhibit high hydroxyl radical scavenging capacity, metal ion chelation ability, and iron ion reduction capacity [89]. These findings collectively demonstrate that ginseng exosomes possess potent antioxidant properties. Overall, ginseng exosomes exhibit significant anti-cancer, anti-inflammatory, neuroprotective, and antioxidant activities, making them a promising new carrier for disease prevention and health foods (Figure 5).

#### 3.2.4. Metabolic Regulatory Effects

Metabolic regulation is a process that controls the synthesis, decomposition and energy conversion of substances through neural, humoral and other mechanisms to adapt to changes in the internal and external environment. This process is closely related to the function of biological barriers [90]. Biological barriers create a stable physical and chemical environment for metabolic activities by maintaining “internal environmental homeostasis”. For instance, the barrier formed by the tight connection of intestinal mucosal epithelial cells (IECs) can prevent harmful substances such as bacteria and toxins in the intestines from entering the bloodstream, thus avoiding the interference of these substances with the normal functions of metabolic organs such as the liver and pancreas (insulin secretion and glycolipid metabolism). In addition, the metabolic state can also directly affect the structural integrity and functional activity of the barrier. Studies have shown that obesity can cause abnormal fat metabolism, leading to chronic inflammation and damage the vascular endothelial barrier (increasing the risk of cardiovascular diseases) [91]. The hyperglycemic state of diabetic patients can damage the skin barrier, causing wound infections that are difficult to recover from. Ginseng exosomes suppress activity within the pentose phosphate pathway (PPP) in lung cancer models, disrupting tumor cell energy metabolism and attenuating epithelial–mesenchymal transition (EMT) [92]. These effects underscore the regulatory role of ginseng exosomes in modulating tumor metabolism.

### 3.3. Application Strategies for Ginseng Exosomes in Related Fields

Ginseng exosomes demonstrate promising potential as drug delivery carriers and in food development, effectively enhancing the bioavailability of foods [93]. Simultaneously, ginseng exosomes exhibit significant therapeutic efficacy in treating cancer, immune system disorders, and other conditions, providing scientific rationale for functional food research and development [94]. Research indicated that leveraging ginseng exosomes as core technology enables them to maintain activity in acidic foods like fruit juices and carbonated beverages while masking the characteristic bitterness of ginseng. This effectively addresses the challenge of balancing efficacy and palatability in functional foods, thereby enhancing the value of health supplements and promoting overall human wellness [86]. Compared to other plant exosomes [95,96], ginseng exosomes offer additional advantages of high bioavailability, pronounced effects, and precise targeting. Rich in bioactive compounds such as ginsenosides and specific small RNAs, these components enhance their stability, bioavailability, and precise gene regulation capabilities within target cells, holding promise for advancing the development of functional foods. Nevertheless, the mechanisms and kinetics underlying ginseng exosome delivery remain unclear. Large-scale extraction and purification processes are both challenging and costly, necessitating the development of efficient extraction and purification techniques to elucidate their practical application value.

## 4. The Current Application Status of Ginseng Proteins

Proteins are crucial natural active ingredients in *P. ginseng*, possessing irreplaceable pharmacological effects. In recent years, advances in biotechnology and proteomics have significantly heightened scientific interest in ginseng protein research [91]. These proteins exhibit a broad spectrum of biological activities, including immune system regulation, antioxidant effects, and anti-inflammatory effects. Furthermore, ginseng protein exhibits enhanced digestibility and low allergenicity following enzymatic hydrolysis. Its diverse bioactive peptides, such as ginseng hypotensive peptides and antioxidant peptides, effectively assist in regulating blood pressure, boosting immunity, scavenging free radicals, and promoting cellular growth and repair [97]. Based on the aforementioned research findings, ginseng protein demonstrates significant potential for application as a nutritional fortifier in functional foods and sports nutrition products. This approach not only supplements high-quality protein but also imparts specific health benefits to products, partially addressing the nutritional needs of diverse populations. These unique biological characteristics have positioned ginseng protein as a focal point in current pharmacological and nutritional research [90]. However, the safety profile, scope of application, human allergenicity, and regulatory data for ginseng protein in functional foods and drug development remain incomplete and warrant further investigation. Therefore, this section summarizes the current research status of ginseng protein, primarily covering its composition, biological functions, and specific application areas, aiming to provide a foundation for in-depth studies on ginseng protein.

### 4.1. The Composition and Extraction Process of Ginseng Protein

Ginseng proteins primarily originate from the root of ginseng, exhibiting a highly complex internal structure. Over 40 distinct types have been identified to date, categorized into RNA-like proteases, ribonuclease-like proteins, and chitin-like proteins. Among these, RNA-like proteases constitute the predominant protein component of *P. ginseng* [98]. Ginseng proteins are primarily composed of essential and non-essential amino acids. However, their unique peptide composition and spatial structure confer significant biological activity. The specific arrangement of amino acids determines the functions and characteristics of ginseng proteins. With advances in biotechnology, ginseng proteins can be extracted using neutral buffer extraction, rapid solvent extraction, and sulfate fractionation precipitation methods. However, the extraction method varies for each specific protein type, closely related to its physiological function (Table 2). As research deepens, the roles of these bioactive components are increasingly recognized, revealing their potential benefits for animal and human health.

### 4.2. Biological Functions of Ginseng Protein

Modern research has discovered that specific proteins extracted from ginseng can stimulate macrophage activity, enhancing their phagocytic capacity and efficiency in eliminating pathogens, thereby significantly boosting immune system function. Beyond regulating immune function, ginseng proteins exhibit significant anti-inflammatory and antibacterial activities [99,107,108]. By suppressing inflammatory responses and mitigating the damage caused by inflammation to the body, they achieve therapeutic effects for various inflammatory diseases. Cole et al. [109] employed a combined proteomics and peptidomics analysis to investigate the antibacterial activity of ginseng total proteins and their hydrolysate products. Results demonstrated that ginseng proteins and peptides exhibit significant antimicrobial properties when subjected to simulated gastrointestinal digestion and microbial community changes. This discovery deepens our understanding of ginseng’s biological activity and provides crucial insights for developing novel antimicrobial agents.

Research has revealed that glycoproteins and certain functionally specialized proteins in ginseng exhibit remarkable antioxidant properties, effectively scavenging free radicals and shielding cells from oxidative damage [110,111]. Under specific conditions, they can also delay the aging process and enhance the survival capacity of aging cells under oxidative stress, thereby maintaining their normal functions. Consequently, ginseng’s total proteins and glycoproteins contribute to delaying aging and promoting cellular health by alleviating oxidative stress and eliminating ROS aggregation [112,113].

Research has confirmed that ginseng proteins possess multiple pharmacological effects, including antitumor, anti-radiation [114], anti-viral, antioxidant, anti-fatigue, and neuroprotective properties [115,116,117]. They effectively improve cognitive impairment, thereby exerting therapeutic effects on Alzheimer’s disease [118]. The aforementioned studies demonstrate that ginseng protein may exert neuroprotective effects through mechanisms such as regulating neurotransmitters, further supporting its potential application in preventing and treating neurological diseases. In addition, some studies have mentioned that ginseng protein may alleviate the symptoms related to hyperlipidemia by regulating insulin sensitivity, lipid metabolism, and other related pathways.

### 4.3. Application Strategies for Ginseng Proteins in Related Fields

**Figure 6:**
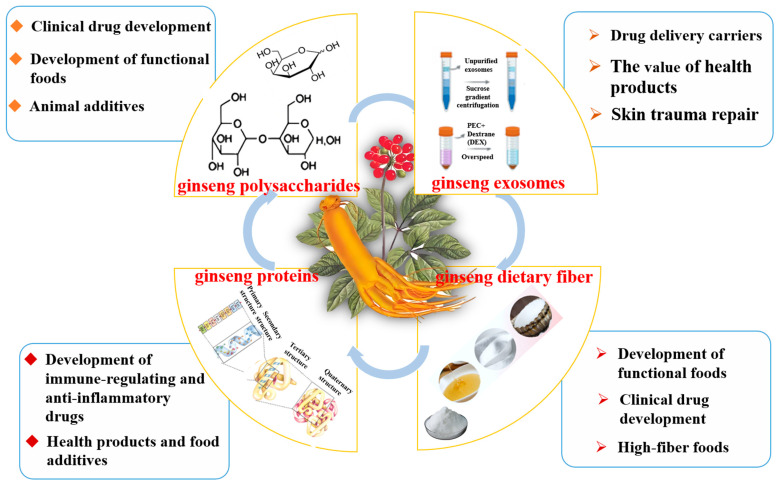
Schematic diagram of the overall application potential of biomacromolecules in *P. ginseng*.

The diverse biological functions of ginseng proteins provide a strong basis for promoting their application in food-related fields and make them an ideal choice for developing novel immunomodulators and anti-inflammatory drugs. These functional proteins can serve as health supplements and food additives, endowing products with specific health benefits to enhance quality of life while meeting the nutritional needs of diverse populations and boosting the nutritional value of foods [119]. With the continuous application of proteomics technology, integrating mass spectrometry with bioinformatics has become a key focus in modern research, significantly advancing the structural elucidation and quantitative analysis of multiple proteins in ginseng. In summary, an in-depth analysis of the composition and functional effects of ginseng proteins will clarify their biological characteristics, providing a solid scientific basis for pharmacological and nutritional applications. This not only enriches the nutritional profile of modern foods but also enhances the added value of functional products by leveraging ginseng’s traditional efficacy, aligning with contemporary consumer trends toward “natural, healthy, and functional” food choices. With advancing research, ginseng protein holds promise to drive the development of novel therapeutic approaches, propel progress in the health industry, and contribute to improving public health (Figure 6).

## 5. The Current Application Status of Ginseng Dietary Fiber

Ginseng dietary fiber is a functional dietary fiber extracted from ginseng residue as the core raw material through processes such as composite enzymatic methods, microbial fermentation, or ultrasound-assisted enzymatic hydrolysis. As a representative product of ginseng deep processing, it is primarily categorized into soluble dietary fiber (SDF) and insoluble dietary fiber (IDF). Rich in carbohydrates, proteins, glucuronic acid, and various amino acids, it is a natural macromolecular substance with high nutritional value and low caloric content. Research confirms that ginseng dietary fiber effectively regulates gut microbiota composition. Its water-soluble components exhibit significant lipid-lowering effects and anti-aging activity, while also demonstrating glucose adsorption, antioxidant properties, and the ability to improve the intestinal microenvironment [120,121]. In recent years, researchers have recognized the immense application value of ginseng dietary fiber. This review section covers the diversity, structural characteristics, functional activities, and other aspects of ginseng dietary fiber [122].

### 5.1. Composition and Classification of Ginseng Dietary Fiber

In the ginseng-related industries, a significant amount of dietary fiber primarily derives from the industrial residues generated during ginseng processing [123,124]. Non-medicinal parts of ginseng are often regarded as byproducts of the ginseng industry chain and serve as a significant source for extracting ginseng dietary fiber. Ginseng dietary fiber possesses a unique porous structure and polysaccharide functional groups, endowing it with outstanding physicochemical properties. This foundation supports its potential biological functions and applications within the food sector. Specifically, ginseng dietary fiber primarily consists of cellulose, hemicellulose, lignin, acidic heteropolysaccharides, and minerals. Research indicates that water-soluble fibers (such as acidic heteropolysaccharides) exhibit excellent solubility, significantly enhancing bioavailability and promoting human absorption and utilization. Conversely, insoluble dietary fiber (such as cellulose) plays a crucial role in stimulating intestinal motility and improving digestive health [125]. This complements the functions of soluble fiber, offering diverse application value in fields like food and pharmaceuticals.

### 5.2. Biological Function of Ginseng Dietary Fiber

Research has confirmed that ginseng dietary fiber possesses unique therapeutic effects in improving intestinal mucosal barrier function and modulating the composition and function of the gut microbiota [126]. This is primarily reflected in enhanced intestinal barrier integrity and elevated levels of SCFAs, which serve as energy sources for maintaining homeostasis within the intestinal mucosal epithelial cells [127]. These SCFAs are important energy sources for intestinal epithelial cells and play a key role in maintaining intestinal health and function [128]. In particular, water-soluble dietary fibers exhibit significant prebiotic effects, effectively regulating the gut microbiota, improving the intestinal microecology, and promoting the growth of bifidobacterium and lactobacillius [122,129]. Additionally, water-soluble fiber increases the concentration of SCFAs in stool, further promoting gastrointestinal health [130]. Meanwhile, insoluble dietary fiber possesses water-absorbing properties that effectively enhance water retention within the intestines and stimulate intestinal peristalsis, thereby effectively preventing constipation. Therefore, increasing dietary fiber intake, particularly from foods rich in ginseng components, plays a significant positive role in maintaining and improving intestinal health.

Ginseng dietary fiber can regulate glucose and lipid metabolism as well as insulin resistance, thereby helping to improve the health in metabolic diseases. SDF slows digestion primarily by delaying the rate at which glucose enters the bloodstream, thereby stabilizing blood sugar levels [131,132]. This effect enhances glucose tolerance and significantly reduces the risk of type 2 diabetes. Particularly, the regulation of blood sugar response by dietary fiber further lowers diabetes risk [133]. Additionally, research indicates that ginseng dietary fiber possesses certain blood pressure-lowering effects. Its primary mechanism lies in the reversible ion exchange between ginseng dietary fiber and sodium ions within the gastrointestinal tract, which promotes the excretion of sodium from the body, thereby achieving an auxiliary effect in regulating blood pressure [134]. This mechanism indicated that ginseng dietary fiber possesses significant biological functions in preventing hypertension and its associated cardiovascular complications.

Furthermore, research indicated that ginseng dietary fiber enhances the total antioxidant capacity of serum [135] and lowers the concentration of inflammatory markers associated with various pathological conditions [136]. These antioxidant and anti-inflammatory effects make ginseng dietary fiber beneficial for improving metabolic health and regulating immune responses. In addition to these biological activities, ginseng dietary fiber increases the levels of insulin-like growth factors (IGF-1 and IGF-2) and immunoglobulins (IgA, IgM, and IgG), thereby enhancing overall immune function [129]. These factors play crucial roles in body growth, tissue repair, and immune responses, underscoring the importance of ginseng dietary fiber in promoting overall health.

### 5.3. Application Strategies for Ginseng Dietary in Related Fields

Previous studies have conclusively demonstrated that ginseng dietary fiber effectively regulates intestinal health and possesses biological functions that lower blood sugar and blood lipids. It can be used as an additive in high-fiber foods or as a dietary supplement in modern foods to enhance flavor and nutritional value [137]. Notably, consuming ginseng dietary fiber induces a feeling of fullness, which aids in controlling food intake. In the functional food sector, ginseng dietary fiber demonstrates significant application potential. As a dietary supplement, it helps improve digestive health, enhance quality of life, and boost the body’s immunity. This characteristic not only effectively increases the added value of ginseng products but also promotes the integration of ginseng medicine with health and nutrition.

Additionally, the waste liquid and residue generated during the extraction of ginseng dietary fiber can be reprocessed to produce bio-based biodegradable food packaging materials. These materials combine freshness preservation and antimicrobial properties while being naturally degradable, offering an alternative to traditional plastic packaging. This contributes to the food industry’s “carbon reduction” goals and aligns with global green development principles.

## 6. Safety Assessment of Ginseng Biomacromolecules

To date, no reports of toxicity associated with ginseng biomacromolecules have been documented; however, safety assessment remains critical for the development and application of ginseng macromolecules. Existing research indicates that ginseng polysaccharides, proteins, and dietary fiber exhibit minimal side effects, significant biological activity, well-defined targets, and relatively high safety profiles. These components show promising potential in experimental animal studies and functional product development, though clinical research remains limited. However, as clinical practice and research advance, while acknowledging their therapeutic effects, attention must be paid to polysaccharide purity and variations in administration routes. During use, the primary principle should be avoiding excessive dosing while fully considering individual variations, particularly for populations with unique constitutions and special groups like the elderly and children. Medications must also be rationally combined based on the principle of syndrome differentiation and treatment. Risk prevention and control should be implemented throughout the usage process, organically integrating drug use with early warning monitoring and adaptive assessment of herbal toxicity to establish a scientific, standardized evaluation pathway and system.

## 7. Conclusions and Prospects

With technological innovations in the food industry and evolving demands in the consumer market, modern research on ginseng biomacromolecules has become a focal point. Among these, ginseng polysaccharides, proteins, exosomes, and dietary fiber hold significant importance in the pharmacological characteristics of ginseng. Particularly, structural analysis, biological functions, and application potential are key areas for in-depth exploration of ginseng’s functional factors. These biomacromolecules play a pivotal role in food processing, exhibiting diverse applications and emerging as core ingredients in functional food development. Ginseng biomacromolecules offer multiple benefits, including immune enhancement, sleep improvement, anti-diabetic effects, gut microbiota regulation, and fatigue relief. Their comprehensive incorporation into foods can elevate nutritional value and meet consumer demand for health-promoting products. Simultaneously, ginseng biomacromolecules also can be optimized through scientific formulation and advanced processing based on physiological indicators and metabolic characteristics of different populations. By dynamically adjusting process parameters such as temperature and pH during food processing, the activity of biomacromolecules is maximally preserved. This addresses the technical challenge of “activity loss versus efficiency imbalance” in food processing, enabling the development of personalized foods. For instance, a sleep-aid meal replacement shakes containing high-activity ginseng polysaccharides and γ-aminobutyric acid (GABA) can be tailored for individuals experiencing immune suppression due to sleep deprivation. Similarly, a sports recovery drink combining ginseng peptides with branched-chain amino acids (BCAAs) can be designed for fitness enthusiasts, leveraging the rapid absorption of peptides to shorten muscle repair cycles. It Is noteworthily that despite significant achievements in previous research on ginseng biomacromolecules, further exploration is needed to clarify the relationship between their molecular structures and pharmacological activities, as well as their application value in the food sector, thereby defining their development potential. Furthermore, in ginseng-related products, the dosage of ginseng is strictly regulated. Excessive daily intake by adults may lead to nervous system hyperactivity symptoms such as excitement, anxiety, and insomnia. This restriction limits the addition of ginseng biomacromolecules in such foods and the full expression of their product efficacy.

In the future, research should focus on elucidating the specific mechanisms of action and nutritional value of these biomacromolecules. Simultaneously, to effectively harness its health benefits, efforts should be made to explore how modern biotechnology can enhance its extraction and application levels, addressing technical challenges such as the susceptibility of biomacromolecules to degradation, low bioavailability, and diminished efficacy. For instance, researchers could establish a more comprehensive scientific foundation for studying ginseng and its bioactive molecules through interdisciplinary collaboration and the integration of modern biotechnology with advanced chemical analysis techniques. In particular, given the challenges we face, including the low yield of plant-derived exosomes, the heterogeneity of pectin structure affecting reproducibility, and the limited clinical data on protein safety, future work should focus on scalable production methods and standardized characterization protocols.

In conclusion, research on ginseng polysaccharides, pectin, exosomes, dietary fiber, and proteins has achieved significant progress in food-related fields. This has promoted the efficient utilization of the entire ginseng plant, enhanced resource utilization rates, prevented resource wastage, increased the added value of ginseng products, and driven the transformation of the ginseng industry from traditional processing to deep development, thereby boosting industrial competitiveness. We anticipate that future research will further unlock the potential value of these bioactive molecules, driving the in-depth development and application of ginseng and its related products. This will enhance market recognition for functional foods and meet consumers’ personalized needs.

## Figures and Tables

**Table 1:** An overall overview of the structure and function of ginseng polysaccharides and its possible mechanism.

Ginseng Polysaccharides Extract	Structural Features	Model/Experiment Types	Biological Activity	Mechanism	References
water-soluble ginseng polysaccharides extract (WGPE), Ginseng polysaccharide extract (GPE), WGPA	RG-I, HG, AG (The acidic polysaccharides isolated from the roots of *P. ginseng* all contain L-Ara, D-Gal, L-Rha, D-GalA and D-GlcA)	Male ICR mice	Immunoregulatory	Stimulate the proliferation of T and B lymphocytes	[21,22]
water-soluble ginseng polysaccharides (GPW-1), GPR-1, GPS-1	RG, HG, AG	Male ICR mice	Hypoglycemic	Increase liver glycogen and insulin levels while reducing TG and TC levels	[20]
GPW-1, GPR-1, GPS-1	RG, HG, AG	Male ICR mice	Antioxidant	Increase the content of non-enzymatic antioxidants and enhance the activity of antioxidant enzymes	[20]
WGPA-3-RG, WGPA-3-HG	RG-I, HG (α-1,2-linked Rha and α-1,4-linked Gal A units)	L-929 cells, HT-29 cells	Anti-tumor	Inhibits cell adhesion and tumor cell migration; induces apoptosis by activating caspase-3	[23]
ginseng polysaccharides extract (PGAP)	HG (α-1,4-glycosidic bonds)	BALB/c mice peritoneal Macrophages; C57BL/6 mice splenocytes	Anti-tumor	Induction of cell cycle arrest and apoptosis and its synergistic effect with paclitaxel	[24,25]
RG-I-4	RG-I, AG-I (α-1,2-linked Rha and α-1,4-linked Gal A units)	Galectin-3–mediated HT-29 cell adhesion	Anti-tumor	binding and inhibition of galectin-3	[26]

**Table 2:** Functional classification of ginseng proteins.

Source	Ginseng Protein type	Quantity Currently Isolated	Relative Molecular Mass	Activity	References
The roots of *P. ginseng*	RNA-like endoribonuclease protein	1	2.8 × 10^4^	Antifungal, antiviral, and transcriptional inhibitory activities	[99,100]
The roots of *P. ginseng*	Ribonuclease	2	2.7 × 10^4^ and 2.9 × 10^4^	Antifungal, antiviral and proliferation-inhibiting activities	[101]
the flower buds of *P. ginseng ginseng*	Ribonuclease	1	2.3 × 10^4^	No antifungal, antiviral, or proliferation-inhibiting activity	[102]
Ginseng callus	Ribonuclease	2	1.8 × 10^4^	Not clear statement	[103]
The roots of *P. ginseng*	Saponin synthesis-related enzymes	1	5.9 × 10^4^	Hydrolyzing Rg3 yields the anti-cancer substance Rh2	[104]
The roots of *P. ginseng*	Chitin-like protein	1	1.5 × 10^4^	Antifungal	[105]
The roots of *P. ginseng*	Xylanase	1	1.5 × 10^4^	Artificial immunodeficiency virus transcriptional suppressor activity	[106]

## Data Availability

No new data were created or analyzed in this study. Data sharing is not applicable to this article.

## Abbreviations

The following abbreviations are used in this manuscript:

AG	arabinogalactan
Ara	arabinose
AX	arabinoxylan
DE	degree of esterification
EAE	experimental autoimmune encephalomyelitis
Gal A	galacturonic acid
Gal	galactose
GAL-3	galectin-3
GDNPs	ginseng-derived nanoparticles
GENs	ginseng extract nanoparticles
Glu A	glucuronic acid
HG	homogalacturonan
HT-29	human colorectal adenocarcinoma cells
IgA/IgM/IgG	immunoglobulin A/M/G
IGF-1/2	insulin-like growth factor-1/2
IL-1α/β	interleukin-1 alpha/beta
IL-6	interleukin-6
MDA	malondialdehyde
NAFLD	non-alcoholic fatty liver disease
RG	rhamnogalacturonan
RG-I	rhamnogalacturonan I
RG-II	rhamnogalacturonan II
Rha	rhamnose
ROS	reactive oxygen species
SCFAs	short-chain fatty acids
SDF	soluble dietary fiber
TAMs	tumor-associated macrophages
IDF	insoluble dietary fiber
TCM	traditional Chinese medicine
TNF-α	tumor necrosis factor-alpha
TOR	target of rapamycin
UC	ulcerative colitis
WGPA	water-soluble ginseng pectin acid
